# Supplementary figures and images for: Vertical transmission of attaching and invasive E. coli from the dam to neonatal mice predisposes to more severe colitis following exposure to a colitic insult later in life
Source: PLoS One. 2022 Apr 5;17(4):e0266005. doi: 10.1371/journal.pone.0266005 (PMC8982877; doi:10.1371/journal.pone.0266005)

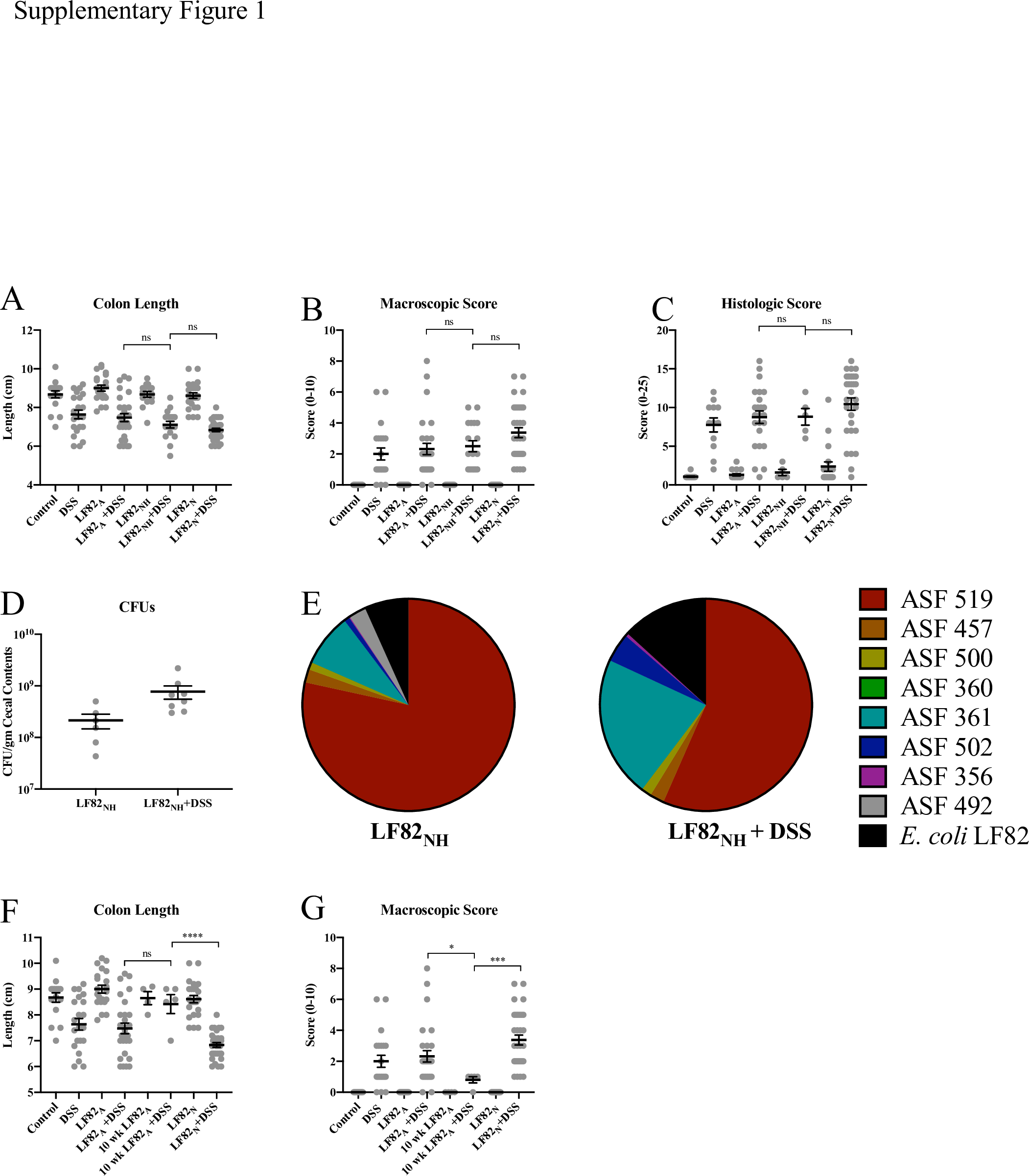

Supplement: S1 Fig — Mice were horizontally infected as neonates (LF82NH) to assess maternal influence on the effects observed following colonization with E. coli LF82. Mice were evaluated at the time of necropsy for parameters of disease including colon length (A) and macroscopic colitis score (B). Additionally, proximal colon was fixed, sectioned and stained with hematoxylin and eosin and blindly scored by a pathologist for histopathologic parameters of inflammation (C). The cecal load of E. coli LF82 was enumerated by bacteriological culture (D) and 16s rRNA gene amplicon sequencing was performed on DNA recovered from fecal samples to assess relative microbial abundance (E). To mimic the length of time that the neonates were colonized with E. coli LF82, a separate set of adult mice evaluated at 10 weeks post-colonization (10 wk LF82A). Mice were scored at the time of necropsy for parameters of disease including colon length (F) and macroscopic colitis score (G). Shared letters between different groups indicate no statistical significance with an ANOVA or Kruskal-Wallis with multiple group comparisons test; ns indicates no statistical significance based on a direct comparison with a t or Mann-Whitney test. A and B: Control n = 17, DSS n = 21, LF82A n = 21, LF82A + DSS n = 28, LF82NH n = 15, LF82NH + DSS n = 18, LF82N n = 24, LF82N + DSS n = 32; C: Control n = 13, DSS n = 12, LF82A n = 16, LF82A + DSS n = 24, LF82NH n = 5, LF82NH + DSS n = 5, LF82N n = 19, LF82N + DSS n = 29; D and E: LF82NH n = 6, LF82NH + DSS n = 8; A and B: Control n = 17, DSS n = 21, LF82A n = 21, LF82A + DSS n = 28, 10 wk LF82A n = 4, 10 wk LF82A + DSS n = 5, LF82N n = 24, LF82N + DSS n = 32. (TIFF) [file pone.0266005.s001.tiff]

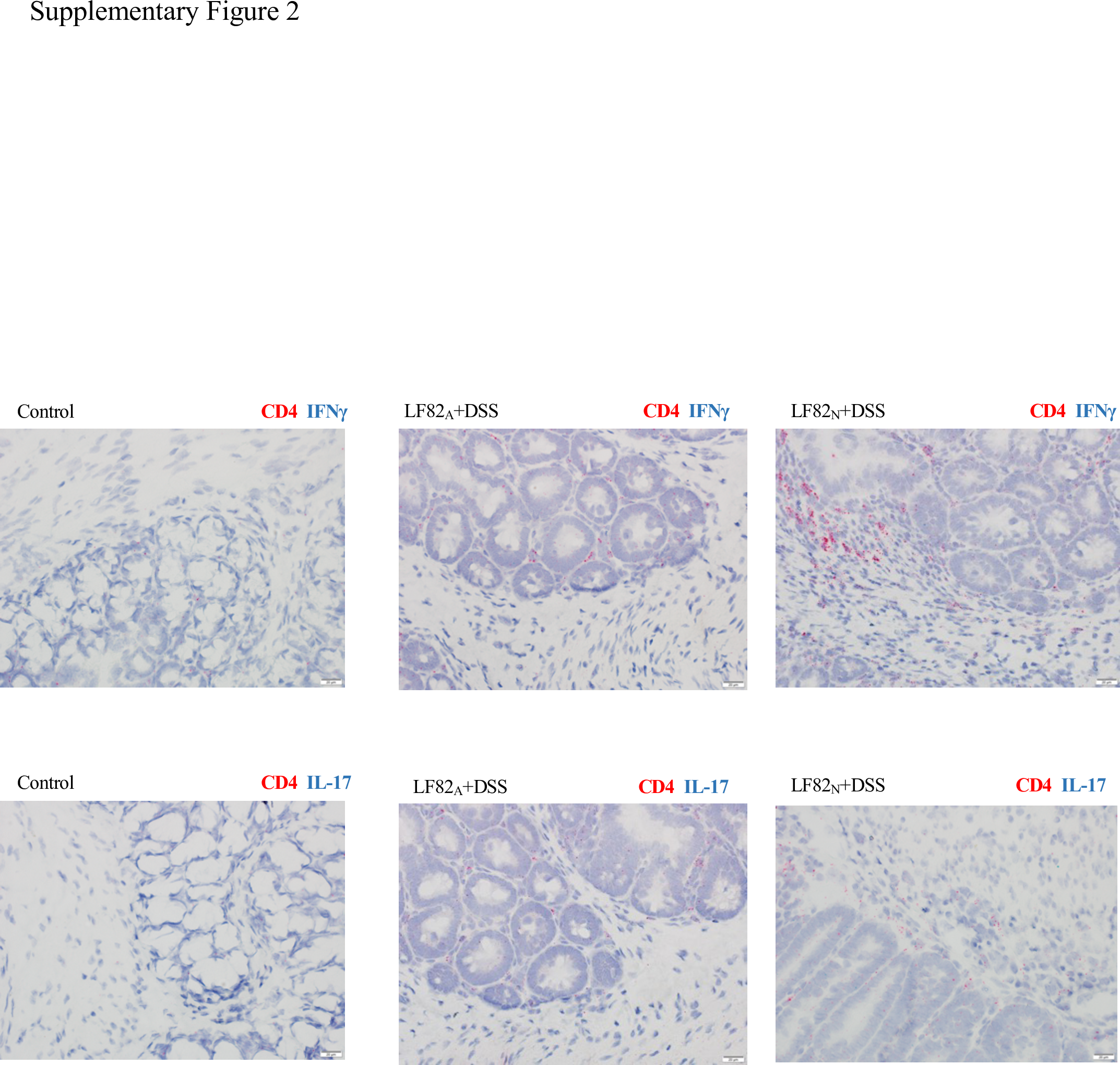

Supplement: S2 Fig — IL-17, IFNγ, and CD4 mRNA was labeled with a duplex RNAscope® assay. Representative photomicrographs demonstrating dual labeling of CD4- (red) and cytokine-specific (green) mRNA in multiple experimental groups (scale bar = 20 μm). (TIFF) [file pone.0266005.s002.tiff]
