# Supplementary material for: Vertical transmission of attaching and invasive E. coli from the dam to neonatal mice predisposes to more severe colitis following exposure to a colitic insult later in life
Source: PLoS One. 2022 Apr 5;17(4):e0266005. doi: 10.1371/journal.pone.0266005 (PMC8982877; doi:10.1371/journal.pone.0266005)
Supplement: S3 Fig — (PDF) [file pone.0266005.s003.pdf]

The Mixed Procedure

Model Information	
**Data Set**	WORK.MYDATA_COLON
**Dependent Variable**	colon
**Covariance Structure**	Diagonal
**Estimation Method**	REML
**Residual Variance Method**	Profile
**Fixed Effects SE Method**	Model-Based
**Degrees of Freedom Method**	Residual

Class Level Information		
**Class**	**Levels**	**Values**
**DSS**	2	0 1
**group**	3	G0 G1 control

Dimensions	
**Covariance Parameters**	1
**Columns in X**	12
**Columns in Z**	0
**Subjects**	1
**Max Obs per Subject**	143

Number of Observations	
**Number of Observations Read**	143
**Number of Observations Used**	143
**Number of Observations Not Used**	0

Covariance Parameter Estimates	
**Cov Parm**	**Estimate**
**Residual**	0.6639

Fit Statistics	
**-2 Res Log Likelihood**	351.6
**AIC (Smaller is Better)**	353.6
**AICC (Smaller is Better)**	353.6
**BIC (Smaller is Better)**	356.5

The Mixed Procedure

Solution for Fixed Effects							
**Effect**	**group**	**DSS**	**Estimate**	**Standard Error**	**DF**	**t Value**	**Pr > |t|**
**Intercept**			7.6381	0.1778	137	42.96	<.0001
**DSS**		0	1.0384	0.2658	137	3.91	0.0001
**DSS**		1	0	.	.	.	.
**group**	G0		-0.1595	0.2352	137	-0.68	0.4988
**group**	G1		-0.8006	0.2288	137	-3.50	0.0006
**group**	control		0	.	.	.	.
**DSS*group**	G0	0	0.4831	0.3549	137	1.36	0.1758
**DSS*group**	G1	0	0.7366	0.3451	137	2.13	0.0346
**DSS*group**	control	0	0	.	.	.	.
**DSS*group**	G0	1	0	.	.	.	.
**DSS*group**	G1	1	0	.	.	.	.
**DSS*group**	control	1	0	.	.	.	.

Type 3 Tests of Fixed Effects				
**Effect**	**Num DF**	**Den DF**	**F Value**	**Pr > F**
**DSS**	1	137	107.75	<.0001
**group**	2	137	5.87	0.0036
**DSS*group**	2	137	2.29	0.1050

Estimates								
**Label**	**Estimate**	**Standard Error**	**DF**	**t Value**	**Pr > |t|**	**Alpha**	**Lower**	**Upper**
**Effect of DSS in G0**	1.5214	0.2352	137	6.47	<.0001	0.05	1.0563	1.9865
**Effect of DSS in G1**	1.7750	0.2200	137	8.07	<.0001	0.05	1.3399	2.2101
**Effect of DSS in control**	1.0384	0.2658	137	3.91	0.0001	0.05	0.5127	1.5640
**DSS-noDSS in G0 and G1**	-0.2536	0.3221	137	-0.79	0.4325	0.05	-0.8904	0.3833
**DSS-noDSS in G0 and control**	0.4831	0.3549	137	1.36	0.1758	0.05	-0.2188	1.1849
**DSS-noDSS in G1 and control**	0.7366	0.3451	137	2.13	0.0346	0.05	0.05428	1.4190
**Generation diff in DSS**	0.6411	0.2108	137	3.04	0.0028	0.05	0.2241	1.0580
**Generation diff in noDSS**	0.3875	0.2435	137	1.59	0.1138	0.05	-0.09393	0.8689

The Mixed Procedure

Contrasts				
Label	Num DF	Den DF	F Value	Pr > F
DSS-noDSS in G0 and G1	1	137	0.62	0.4325
DSS-noDSS in G0 and control	1	137	1.85	0.1758
DSS-noDSS in G1 and control	1	137	4.56	0.0346

Least Squares Means						
Effect	DSS	Estimate	Standard Error	DF	t Value	Pr > |t|
**DSS**	0	8.7630	0.1045	137	83.84	<.0001
**DSS**	1	7.3181	0.09193	137	79.60	<.0001

Differences of Least Squares Means							
Effect	DSS	_DSS	Estimate	Standard Error	DF	t Value	Pr > |t|
**DSS**	0	1	1.4449	0.1392	137	10.38	<.0001

The Mixed Procedure

Model Information	
Data Set	WORK.MYDATA
Dependent Variable	colon
Covariance Structure	Diagonal
Estimation Method	REML
Residual Variance Method	Profile
Fixed Effects SE Method	Model-Based
Degrees of Freedom Method	Residual

Class Level Information		
Class	Levels	Values
DSS	2	0 1
group	3	G0 G1 control

Dimensions	
Covariance Parameters	1
Columns in X	12
Columns in Z	0
Subjects	1
Max Obs per Subject	143

Number of Observations	
Number of Observations Read	143
Number of Observations Used	143
Number of Observations Not Used	0

Covariance Parameter Estimates	
Cov Parm	Estimate
Residual	0.6639

Fit Statistics	
-2 Res Log Likelihood	351.6
AIC (Smaller is Better)	353.6
AICC (Smaller is Better)	353.6
BIC (Smaller is Better)	356.5

The Mixed Procedure

Solution for Fixed Effects							
**Effect**	**group**	**DSS**	**Estimate**	**Standard Error**	**DF**	**t Value**	**Pr > |t|**
**Intercept**			7.6381	0.1778	137	42.96	<.0001
**DSS**		0	1.0384	0.2658	137	3.91	0.0001
**DSS**		1	0	.	.	.	.
**group**	G0		-0.1595	0.2352	137	-0.68	0.4988
**group**	G1		-0.8006	0.2288	137	-3.50	0.0006
**group**	control		0	.	.	.	.
**DSS*group**	G0	0	0.4831	0.3549	137	1.36	0.1758
**DSS*group**	G1	0	0.7366	0.3451	137	2.13	0.0346
**DSS*group**	control	0	0	.	.	.	.
**DSS*group**	G0	1	0	.	.	.	.
**DSS*group**	G1	1	0	.	.	.	.
**DSS*group**	control	1	0	.	.	.	.

Type 3 Tests of Fixed Effects				
**Effect**	**Num DF**	**Den DF**	**F Value**	**Pr > F**
**DSS**	1	137	107.75	<.0001
**group**	2	137	5.87	0.0036
**DSS*group**	2	137	2.29	0.1050

Estimates								
**Label**	**Estimate**	**Standard Error**	**DF**	**t Value**	**Pr > |t|**	**Alpha**	**Lower**	**Upper**
**Effect of DSS in G0**	1.5214	0.2352	137	6.47	<.0001	0.05	1.0563	1.9865
**Effect of DSS in G1**	1.7750	0.2200	137	8.07	<.0001	0.05	1.3399	2.2101
**Effect of DSS in control**	1.0384	0.2658	137	3.91	0.0001	0.05	0.5127	1.5640
**DSS-noDSS in G0 and G1**	-0.2536	0.3221	137	-0.79	0.4325	0.05	-0.8904	0.3833
**DSS-noDSS in G0 and control**	0.4831	0.3549	137	1.36	0.1758	0.05	-0.2188	1.1849
**DSS-noDSS in G1 and control**	0.7366	0.3451	137	2.13	0.0346	0.05	0.05428	1.4190

Contrasts				
**Label**	**Num DF**	**Den DF**	**F Value**	**Pr > F**
DSS-noDSS in G0 and G1	1	137	0.62	0.4325
DSS-noDSS in G0 and control	1	137	1.85	0.1758
DSS-noDSS in G1 and control	1	137	4.56	0.0346

The Mixed Procedure

Least Squares Means						
**Effect**	**DSS**	**Estimate**	**Standard Error**	**DF**	**t Value**	**Pr > |t|**
**DSS**	0	8.7630	0.1045	137	83.84	<.0001
**DSS**	1	7.3181	0.09193	137	79.60	<.0001

Differences of Least Squares Means							
**Effect**	**DSS**	**_DSS**	**Estimate**	**Standard Error**	**DF**	**t Value**	**Pr > |t|**
**DSS**	0	1	1.4449	0.1392	137	10.38	<.0001
