# Supplementary material for: Vertical transmission of attaching and invasive E. coli from the dam to neonatal mice predisposes to more severe colitis following exposure to a colitic insult later in life
Source: PLoS One. 2022 Apr 5;17(4):e0266005. doi: 10.1371/journal.pone.0266005 (PMC8982877; doi:10.1371/journal.pone.0266005)
Supplement: Attachment — Submitted filename: Response to Reviewers Comments PONE-D-21-36309.docx [file pone.0266005.s004.docx]

On behalf of my co-authors, below, please find our responses to the reviewer’s comments for PONE-D-21-36309.

Respectfully,

Michael J. Wannemuehler, PhD

Professor and Chair

515-294-3534

[mjwannem@iastate.edu](mailto:mjwannem@iastate.edu)

Responses to reviewer’s specific comments:

Reviewer #1:

1. *In Figure 2, although difference between vertically colonized mice and horizontally colonized ones is statistically significant, severity between two groups of mice is marginal at best. Considered that authors have rigorously performed statistical analysis using large number of mice, which was respectable, this reviewer is afraid whether AIEC-colonization during neonate has little or no impact on the mucosal immunity in terms of the severity of DSS-induced colitis. This reviewer proposes that authors mention about a possibility of pathobionts other than AIEC have larger impact on gut homeostasis in the DISCUSSION section.*

We thank the reviewer for this comment and we would like to make the point that no single parameter represented a significant change between the mice that were colonized with AIEC LF82 as neonates versus adults in the absence of a colitic insult (i.e., DSS). As IBD/colitis is a multifactorial disease, it is our contention that one needs to take into account the entirety of the host response. For this reason, the PCA plots (especially Figure 6B) were included to make that point. We can certainly add information to the discussion that other pathobionts, such as *Helicobacter bilis,* alone induce histopathological changes and that colitic lesions can be induced with a lower dose of DSS (1.5 %) that was used in the current work (2 %) (see publications 57, 58, 59). Also, additional text has been added to the Discussion (lines 440 – 447) to provide information about other pathobiont that have been shown to exacerbate colitis or IBD.

2. *Authors clearly showed that vertical colonization with AIEC does not change the mucosal pathology in steady-state. Explain more carefully the possible mechanism associated with the enhanced colitis, including enhanced colon IL-17 mRNA level, in mice colonized as a neonate, please. Again, related to comment 1, a possibility of vertical colonization with AIEC do regulate gut homeostasis in some manner but having little impact on the pathology of DSS-induced colitis could not be excluded.*

We appreciate the Reviewer’s comment and have addressed these concerns as follows. With respect to the elevated IL-17 response, we did detect significantly greater amounts of IL-17 secreted from colon tissue of the neonates colonized with AIEC LF82 following onset of colitis than any of the other groups as shown in Figure 5A. As suggested by the reviewer, colonization alone did not result in an increase in the secretion of IL-17; this only occurred following exposure to the low dose of DSS used in this model. At this time, the mechanism associated with this observation is unclear. However, in a companion manuscript which is in preparation, we have observed that AIEC colonization of neonatal mice increased the mucosal gene expression of multiple pro-inflammatory chemokines/cytokines (e.g., CCL4, CCL5, CXCL14, CXCL5, TNF) that were not upregulated in the mucosa of mice colonized with AIEC LF82 as adults. While we are still analyzing this RNAseq data, it appears that innate immunity was heightened/modified by the colonization of neonates by *E. coli* LF82. Additionally, we have preliminary data indicating that there are more epigenetic changes (i.e., methylation) induced in by the colonization of neonates by *E. coli* LF82 compared to colonization of adult mice.

Answering this query from a different perspective, the RNAscope analysis (Table 1) indicates that a significant portion of the IL-17 and IFNg was produced by CD4-negative cells suggesting a potential role of innate lymphoid cells (ILC1 and ILC 3) in the exacerbated colitis.

Reviewer #2

1. *The authors concluded that the DSS-induced colitis was exacerbated in the LF82N group compared to the LF82A group based on the length of the colon. Although the macroscopic scores were correlated with the length of the colon, histological scores of the colitis between the two groups were not different. The authors need to discuss these points or replace them with more representative ones to support the authors’ conclusion.*

We appreciate the Reviewer’s comment and would like to make the point that our model purposefully utilized a low dose of DSS, that alone induced mild to no clinical of histological inflammation. In this context, parameters of DSS-induced inflammation often measure are less drastic/dramatic than in studies where higher doses (e.g., > 3 %) of DSS are used. We have added a statement to the Materials and Methods section to clarify this point (lines 152 -154). In the context of these studies, we would also make the point that it is the collective nature of the host responses that contribute to the more severe lesions noted as is described in the PCA plots (Fig. 6B). We would also like to make the point that in many respects we are comparing apples and oranges when considering that morphologic changes in colon length that occur over time while histological lesions comprises a snap-shot of both inflammatory and morphologic changes at the time point the tissue was collected.

2. *In Figure 2D, the colonic mucosa in DSS-treated mice seemed to be normal. In general, DSS treatment usually induces focal ulceration of the colonic mucosa along with massive infiltration of mononuclear cells, as observed in the colon of LF82N + DSS. Thus, it is unclear why DSS treatment did not induce such changes. The authors need to explain apparent inconsistent results.*

We appreciate the Reviewer’s comment and apologize for omitting a key aspect of this model. As our goal was to assess the impact of the provocateur on the severity of the colitis and not colitis induced by DSS; therefore, we designed the model to use a low dose of DSS (2 %) that induced little or no disease. We failed to include this point in the manuscript which would leave a reader wondering why the DSS-treatment had not induced colitis. We have amended the manuscript to include this point (lines 152 – 159) which should clarify why the expected inflammation normally associated with DSS exposure was not observed. Furthermore, we would like to emphasized to the Reviewer that exposure to increased doses of DSS in our model do indeed result in more severe macroscopic lesions and histologic inflammation as observed in other published studies.

3. *In Figure 4, it is unclear whether the positive signals actually represent the existence of the indicated bacteria. Why were the positive signals not detected in the lumen of the colon of LF82N mice? Given that LF82N mice harbored E. coli in the colon, the signals of E. coli should be present in the lumen of the colon. The authors should show these results in a more convincing manner.
   What do G0 and G1 mean?*

We appreciate that the Reviewer caught this oversight in the labeling of the panels for this Figure. While transitioning from a previous draft, we failed to adjust the nomenclature referring to the adult mice (G0 generation vs LF82_A_) versus the neonates (G1 generation vs LF82_N_). We have made the corrections to the Figure and uploaded the corrected version.

The bright orange signals (Cy3-labled *E. coli*) in the background of green (FITC – EUB probe) do indeed indicate the presence of AIEC LF82. The numbers of AIEC LF82 in the lumen of the mice, in the absence of DSS, was approximately 3 x 10^8^ per gram contents. Given that the FISH images in Figure 4 were obtained using a 60X objective and using a 5 to 7 µm thick section. The rationale for showing these photomicrographs was to demonstrate whether or not *E. coli* LF82 became more closely associated with the mucus layer or potentially invaded the tissue (i.e., mucosal associated). That said, it was not unexpected that we would observe few *E. coli* LF82 signals when the CFU counts in control mice were less than 10^9^ per gram contents. For the Reviewer’s reference, we include a lower magnification of the cecal contents from a neonate colonized with AIEC LF82 which shows a modestly abundant number of Cy3-labeled AIEC LF82. This image was previously published (Wymore Brand, M., M. J. Wannemuehler, G. J. Phillips, A. Proctor, A-M Overstreet, A. E. Jergens, R. P. Orcutt, and J. G. Fox. 2015. The altered Schaedler flora: Continued applications of a defined murine microbial community. *ILAR* 56:169-178. doi: 10.1093/ilar/ilv012).

4. In Figure 5, there was no significant difference in cytokine production between the control and the DSS-treated groups at least the authors’ experimental conditions. Given that DSS treatment induces severe inflammation and upregulation of many cytokines, the results seem to be surprising. To further substantiate the authors’ findings, the authors need to check the expression of several cytokines of the colon of untreated and DSS-treated mice by qPCR.

We appreciate the Reviewer’s comment and the fundamental answer to the question is that fact that we used a low dose of DSS as explained above in order to avoid the overt inflammation due soley to DSS. Therefore, the DSS-only treated mice developed very mild host responses by design. That said, we will be able to demonstrate in a companion manuscript that, relative to non-treated, control ASF mice, the low dose DSS treatment induced differential gene expression (i.e., p ≤ 0.05) of 146 genes including *CCL4, CCL5, CXCL14, CXCL5,* and *TNF*.
